# High cord blood CCL22/CXCL10 chemokine ratios precede allergic sensitization in early childhood

**DOI:** 10.18632/oncotarget.13374

**Published:** 2016-11-15

**Authors:** Kuo-Wei Yeh, Chih-Yung Chiu, Kuan-Wen Su, Ming-Han Tsai, Man-Chin Hua, Sui-Ling Liao, Shen-Hao Lai, Li-Chen Chen, Tsung-Chieh Yao, Jing-Long Huang

**Affiliations:** ^1^ Division of Allergy, Asthma and Rheumatology, Department of Pediatrics, Chang Gung Memorial Hospital and Chang Gung University College of Medicine, Taoyuan, Taiwan; ^2^ Community Medicine Research Centre, Chang Gung Memorial Hospital, Keelung, Taiwan; ^3^ Department of Pediatrics, Chang Gung Memorial Hospital at Keelung and Chang Gung University College of Medicine, Taoyuan, Taiwan; ^4^ Division of Pediatric Pulmonology, Chang Gung Memorial Hospital, College of Medicine, Chang Gung University, Taoyuan, Taiwan

**Keywords:** allergic sensitization, atopic diseases, CCL22, cord blood, CXCL10, Immunology and Microbiology Section, Immune response, Immunity

## Abstract

Atopic diseases are known to be characterized by a T helper (Th) 2-skewed immunity; however, there are few studies addressing the Th1/Th2 immunity at birth related to the development of atopic diseases in early childhood. We investigated 186 children followed up regularly at the clinic for 4 years in a birth cohort study. The Th1-associated CXC chemokine ligand (CXCL)-10, CXCL11, and the Th2-associated CC chemokine ligand (CCL)-17 and CCL22 were quantified in cord blood by multiplex Luminex kits. Specific immunoglobulin E antibodies against food and inhalant allergens were measured at 6 months as well as 1, 1.5, 2, 3, and 4 years of age. Cord blood CCL22 levels were positively associated with IgE sensitization at age 2, whereas cord blood CXCL10 levels were negatively associated with mite sensitization at age 3. Furthermore, a high cord blood CCL22/CXCL10 chemokine ratio was significantly associated with a higher risk of allergic sensitization at age 3 (OR, 1.02; 95% confidence interval [CI], 1.0051.039; *P* = 0.012). However, cord blood Th1- and Th2-associated chemokines and their ratios were not associated with atopic diseases at different age. Our study indicates that a Th2-skewed immunity at birth may increase risk of allergic sensitization but not of allergic outcomes later in life.

## INTRODUCTION

A Th1/Th2 cytokine imbalance is subsequently associated with numerous human immunological diseases such as atopy and asthma [1]. Atopic disorders are associated with a shift towards Th2-like cytokines (interleukin-4 (IL-4), IL-5 and IL-13) in children promoting the production of immunoglobulin E (IgE) antibodies [2]. Although atopic diseases are characterized by a Th2-dominant immunity, the balance of Th1/Th2 immunity at birth related to the development of atopic diseases during early childhood has not been extensively approached.

The influence of the maternal immunity is reportedly to have long-term effects on the immunity of the offspring [3, 4]. Cord blood represents a bond between mother and child, which provides a unique opportunity to investigate the immunity of the newborn prior to disease onset. The levels of Th1 and Th2 cytokines are low and close to limit of detection in cord blood, but Th1- and Th2-like chemokines are relatively high and have previously been used as important markers for atopic diseases in children [5]. In addition, sensitization to allergen is known to be a significant factor in the development of atopic diseases. Few studies, however, have addressed the predictive value of cord blood Th1- and Th2-like chemokines for allergen sensitization and the risk of developing atopic diseases in early childhood.

The aim of this study was to investigate the relationships between cord blood Th1-associated CXC chemokine ligand (CXCL-10 and CXCL11) and the Th2-associated CC chemokine ligand (CCL-17 and CCL22) and sensitization to food and mite allergens from birth to 4 years of age in children from a birth cohort in the Prediction of Allergies in Taiwanese Children (PATCH) study. The relevance of Th1/Th2 chemokines at birth to the development of atopic diseases during early childhood was also examined.

## RESULTS

### Study population and characteristics

A total of 258 children were initially enrolled, but only 226 (87.6%), 210 (81.4%), 198 (76.7%), and 186 (72.1%) were regularly followed at the clinic for 1, 2, 3, and 4 years, respectively. The subject flow diagram of this birth cohort study was reported previously [6, 7]. Chemokines were examined in a total of 180 cord blood samples from 186 children regularly followed at the clinic for 4-year follow-up period. There was no significant difference in the baseline characteristics of these 180 children and the full 258 children, indicating that the 180 children enrolled could be a representative sample of the entire birth cohort. Levels of cord blood chemokines, total serum IgE, and the prevalence of allergen sensitization and atopic diseases across different years of ages are shown in Table 1.

**Table 1:** Baseline levels of cord blood chemokines, total serum IgE, and prevalence of allergic sensitization and atopic diseases of 180 children for a 4-year follow-up

		Age				
Variable	Birth (Cord blood)	6 Months	1 Years	2 Years	3 Years	4 Years
CCL22, pg/mL	1359.4 (40.9-4636)					
CCL17, pg/mL	714.0 (74.1-9624)					
CXCL10, pg/mL	38.7 (2.0-967)					
CXCL11, pg/mL	3.4 (0.1-21)					
Total serum IgE, kU/L		13.6 (2.0-352)	34.5 (3.0-675)	64.4 (2.0-720)	54.0 (3.7-1363)	48.6(4.3-1254)
IgE levels ≥ 100 kU/L, n (%)		10 (7.8)	29 (22.3)	35 (34.7)	31 (32.6)	32 (36.8)
Allergic sensitization, n (%)						
Food allergen		26 (20.3)	67 (51.5)	68 (67.3)	52 (54.7)	29 (33.3)
Mite allergen		4 (3.1)	11 (8.5)	32 (31.7)	47 (49.5)	43 (49.4)
Food or mite allergens		26 (20.3)	70 (53.8)	72 (71.3)	69 (72.6)	51 (58.6)
Atopic diseases, n (%)						
Eczema		25 (13.9)	22 (12.2)	22 (12.2)	21 (11.7)	20 (11.1)
Allergic Rhinitis		0 (0.0)	11 (6.1)	37 (20.6)	58 (32.2)	63 (35.0)
Asthma (Infantile wheezing if age ≤ 1)		6 (3.3)	16 (8.9)	19 (10.6)	25 (13.9)	27 (15.0)

Data shown are median (range) or number (%) of patients as appropriate. Infantile wheezing is diagnosed as recurrent wheezing during the first year of life.

### Association between cord blood Th1- and Th2-associated chemokines and allergic sensitization

**Figure 1:**
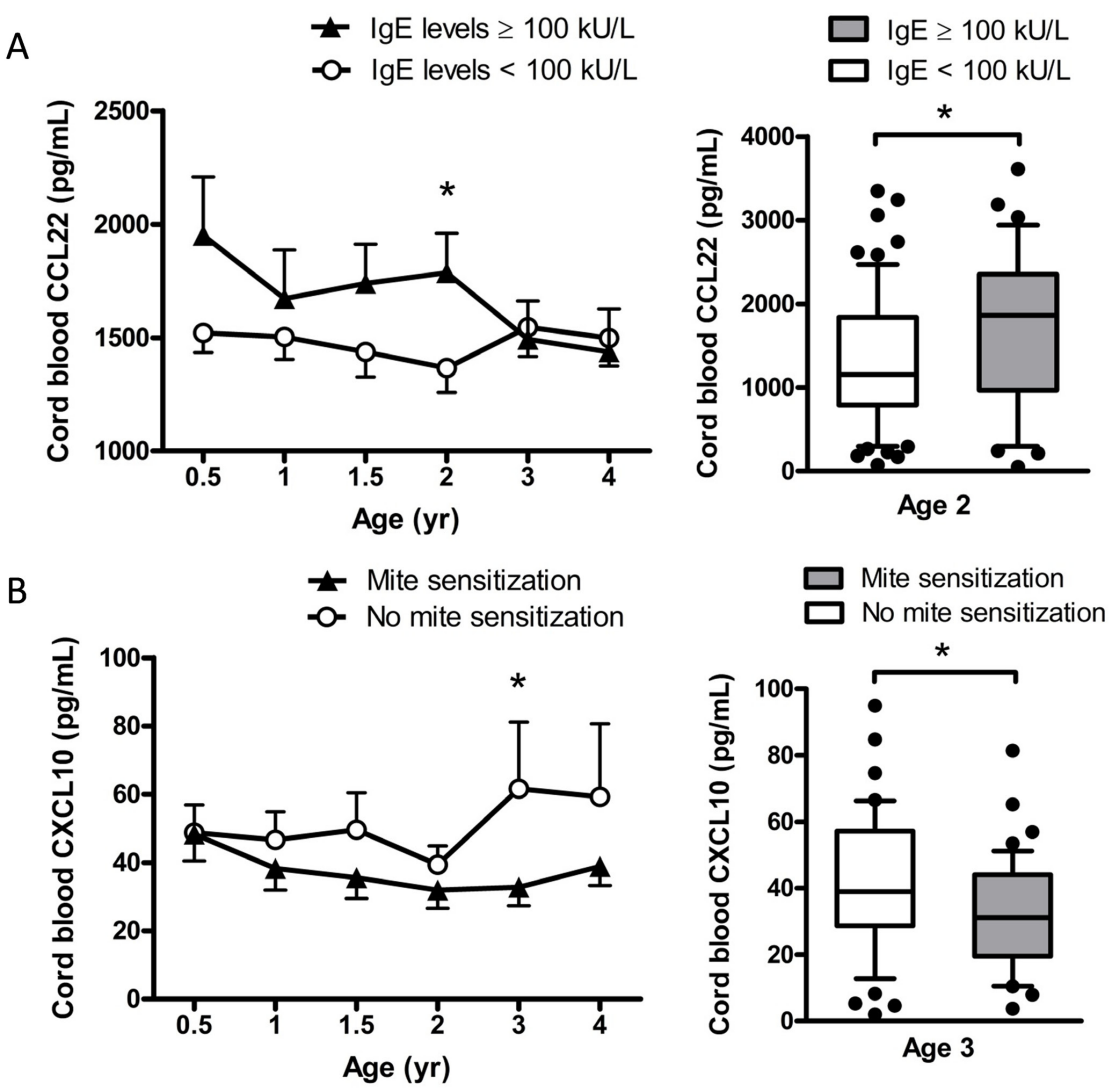
Association of cord blood Th1/Th2-associated chemokines with total serum IgE levels and allergic sensitization at different years of age Comparisons and differences between cord blood CCL22 chemokine and IgE sensitization (IgE levels >= 100 kU/L) **A**., and between cord blood CXCL10 chemokine and mite sensitization **B**. at different years of age. Data shown are mean ± SEM. Box plots showing median and interquartile ranges of cord blood chemokines by subject groups. Dots beyond the bounds of the whiskers denote outliers. *P*-values refer to the comparisons indicated by the marker. *P < 0.05.

Comparisons and differences between cord blood Th1- and Th2-associated chemokines and IgE sensitization (≥ 100 kU/L) and allergen sensitization at different years of age are shown in Figure 1. There was a tendency of high cord blood CCL22 levels associating with high serum total IgE levels (≥ 100 kU/L) before the age of 2 years. By contrast, there was a tendency of low cord blood CXCL10 levels associating with a high prevalence of mite sensitization after age 2. A significant difference and positive association was seen between cord blood CCL22 chemokine levels and IgE sensitization at the age of 2 years (odds ratio [OR], 1.00; 95% confidence interval [CI], 1.001-1.002; *P* = 0.038) (Figure 1A). A significant difference and negative association was seen between cord blood CXCL10 chemokine levels and mite sensitization at the age of 3 years (OR, 0.98; 95% CI, 0.953-0.997; *P* = 0.025) (Figure 1B). A high Th2/Th1 ratio CCL22/CXCL10 was significantly associated with sensitization to any allergens at the age of 3 years (OR, 1.02; 95% CI, 1.005-1.039; *P* = 0.012). There was no association between cord blood CXCL11, CCL17, CCL17/CXCL11 or CCL22/CXCL11 and total serum IgE levels and allergen sensitization.

### Association among cord blood CCL22/CXCL10 chemokine ratios, allergic sensitization and atopic diseases

A ROC curve was generated to determine the sensitivity and specificity of cord blood CCL22/CXCL10 chemokine ratios for discriminating children with and without allergic sensitization. Cord blood CCL22/CXCL10 chemokine ratios had the highest area under the ROC curve (AROC) significantly different from 0.5 at the age of 3 years (AROC = 0.70; 95% CI, 0.592-0.812; *P* = 0.002). The highest combination of sensitivity and specificity was observed with a cut-off level of 36 (60.9% and 69.2%, respectively) for predicting allergic sensitization at age 3. The relationships between high CCL22/CXCL10 ratios (≥ 36) and allergic sensitization and the risk of atopic diseases during early childhood are shown in Table 2. A higher prevalence of sensitization to food or mite allergens was significantly associated with the risk of allergic rhinitis and asthma at ages 3 and 4 years. However, there was no significant association between CCL22/CXCL10 chemokine ratios and atopic diseases at different years of age.

**Table 2:** Association of cord blood CCL22/CXCL10 chemokine ratios and allergic sensitization with the risk of atopic diseases during early childhood

Age	Outcome	Cord blood CCL22/CXCL10 ratio ≥ 36		Sensitization to food or mite allergens at age 3	
		Adjusted OR (95% CI)	*P*	Adjusted OR (95% CI)	*P*
1Y	Eczema	0.77 (0.26-2.25)	0.630	2.84 (0.37-22.09)	0.319
	Allergic rhinitis	0.34 (0.08-1.38)	0.130	5.35 (0.02-132.78)	0.674
	Infantile wheezing	1.14 (0.35-3.72)	0.827	0.99 (0.10-10.27)	0.995
2Y	Eczema	0.96 (0.35-2.64)	0.936	0.99 (0.21-4.77)	0.990
	Allergic rhinitis	0.50 (0.21-1.18)	0.114	3.13 (0.78-12.62)	0.109
	Early-onset asthma	0.57 (0.19-1.72)	0.318	2.74 (0.40-18.74)	0.305
3Y	Eczema	0.67 (0.24-1.88)	0.450	1.46 (0.25-8.40)	0.671
	Allergic rhinitis	0.82 (0.38-1.80)	0.620	**13.36 (2.60-68.62)**	**0.002**
	Asthma	0.81 (0.30-2.16)	0.672	**14.40 (1.30-159.25)**	**0.030**
4Y	Eczema	0.70 (0.25-2.01)	0.513	1.65 (0.26-10.48)	0.596
	Allergic rhinitis	0.71 (0.31-1.61)	0.413	**24.08 (5.29-109.60)**	**0.005**
	Asthma	1.59 (0.59-4.26)	0.361	**7.88 (1.21-51.54)**	**0.031**

Abbreviations: Y, year; CI, confidence interval. OR, odds ratio: adjusted for maternal age at delivery, maternal history of atopy, any passive smoke exposure during pregnancy, any older siblings, household income, gestational age and sex.

All *P*-values < 0.05, which is in bold, are significant.

## DISCUSSION

An immunity skewed toward the Th2-like responses is a characteristic of being atopic. The Th1/Th2 skewing of immunity at birth related to the development of atopic diseases however has not been well assessed in early childhood. This study has demonstrated that high Th2-associated chemokine CCL22 and low Th1-associated chemokine CXCL10 levels in cord blood are associated with raised IgE production and mite sensitization, respectively. A positive, significant association between cord blood CCL22/CXCL10 chemokine ratio and allergen sensitization suggests that an imbalance of Th1/Th2 immunity with Th2 skewing at birth may increase the prevalence of allergic sensitization, which may risk in the development of atopic diseases in early childhood.

Th1- and Th2-type responses and their balances have been postulated to be a predisposing factor for both health and disease development [8]. Th1 and Th2 cytokines are believed to induce the production of the respective chemokines. Th2 chemokines CCL17 and CCL22 are induced by IL-4 and IL-13, whereas Th1 chemokines CXCL10 and CXCL11 are induced by IFN-γ [9, 10]. CXCL10 and CXCL11 levels are reportedly low at birth and increase with age [11]. However, in this study, the cord blood CXCL11 levels were extremely low compared to previous studies. The geographically restricted signatures of INFs and their genetic variations have conferred a selective advantage to the host, which may explain the differences of the expressions of INF-γ-induced CXCL11 in our study [12].

Increased circulating levels of Th2-associated chemokine CCL22 are reportedly associated with allergic symptoms, including atopic dermatitis and asthma [5, 13]. However, high CCL22 levels in cord blood are reportedly associated with IgE production but not the development of atopic diseases as in this study [11, 14]. IgE is a known critical component of allergic diseases. A tendency of positively association between cord blood chemokine CCL22 and IgE sensitization (IgE levels ≥ 100 kU/L) only within the first 2 years of life observed in this study may explain the reports of no association with atopic diseases that usually occur in pre-school children.

Allergic sensitization has consistently been identified as the most important risk factor for atopic diseases [15]. As in this study, allergen sensitization is reportedly related to low levels of Th1-associated chemokines at birth [16]. Furthermore, a tendency of negatively association between cord blood CXCL10 and mite sensitization was observed mainly after the age of 2 years, which is the age at which the prevalence of mite sensitization and asthma increases markedly [7]. These findings suggest that a low Th1 immunity at birth may take part in up-regulation of allergic sensitization, possibly risking the development of atopic diseases later in childhood.

An imbalance in circulating Th1 and Th2 chemokines has been reported to precede allergen sensitization and atopic diseases [5, 14]. In this study, not only high Th2 chemokine (CCL22) and low Th1 chemokine (CXCL10) levels but also a high Th2/Th1 ratio (CCL22/CXCL10) at birth appeared to precede IgE-mediated allergic sensitization in early life. A strong, significant association between allergen sensitization and the risk of developing atopic diseases has been reported as in this study [7]. These results suggest that an imbalance of Th1/Th2 immunity with Th2 skewing at birth may develop allergic sensitization and/or allergic symptoms in early childhood.

A relatively small sample size resulting limited statistical power for the subanalyses is one of the major limitations of this study. Furthermore, this study's relatively short 4-year follow-up period may make the diagnosis of subclinical atopy be missed. A significant strength of this study, however, lies in its longitudinal follow-up at very close intervals. Frequent sequential assessments of allergen sensitization and IgE levels not only provide a systematic observation process but also make the results demonstrated here potentially important.

In conclusion, high levels of Th2-associated chemokines and low levels of Th1-associated chemokines in cord blood appear to precede allergic sensitization in early childhood. An immunity skewed toward the Th2-like responses at birth may increase the risk of allergic sensitization in response to allergens later in life. The determination of a high cord blood Th2/Th1 chemokine ratio (CCL22/CXCL10 ≥ 36) may be useful for predicting the development of allergic sensitization, potentially risking in the development of atopic diseases in early childhood. However, a larger and longer study is required to investigate these associations more comprehensively.

## MATERIALS AND METHODS

### Patients

The PATCH study included subjects from a birth cohort as well as those from several cohorts of school and preschool children [17]. Children who were completed four years of follow-up in a birth cohort study launched at Chang Gung Memorial Hospital (CGMH), Keelung from the year 2007 to 2010 were enrolled. Detailed descriptions of subject recruitment have been reported previously [6]. The details of information regarding demographic data, family atopy history, and the health care history and medical conditions of children were collected. This study was approved by the Ethics Committee of Chang Gung Memorial Hospital (No. 103-6236A3). Written informed consent was obtained from the parents or guardians of all study subjects.

### Evaluation and diagnosis of atopic diseases

Specific symptoms related to atopic diseases were inquired and evaluated by the same pediatric pulmonologist at outpatient clinics for a final diagnosis. As described in our previous study [18], the diagnosis of eczema was defined as a pruritic rash over the face and/or extremities with a chronic relapsing course [19]. Allergic rhinitis was diagnosed as a history of sneezing, nasal congestion, itching, or rhinorrhea or the current use of medication for these symptoms [20]. Asthma was diagnosed as ever having asthma, with the presence of a recurrent wheeze or current use of asthma medication [21]. Infantile wheezing was defined as recurrent wheezing during the first year of life, while early-onset asthma was defined as asthma occurring before the age of 2 years [22].

### Total and allergen-specific serum immunoglobulin E

Total and allergen-specific serum IgE were examined as described in our previous study [18]. Total serum IgE level was measured by ImmunoCAP (Phadia, Uppsala, Sweden) and IgE sensitization was defined as IgE levels ≥ 100 kU/L [23]. Three food allergens (egg white, milk and wheat) and three inhalant allergens (*D. pteronyssinus*, *D. farina* and *C. herbarum*) were measured simultaneously [24]. Allergen-specific IgE was determined using a commercial assay for IgE (ImmunoCAP Phadiatop Infant; Phadia) and allergen sensitization was defined as values ≥ 0.35 kU/L [25].

### Measurement of cord blood Th1- and Th2-associated chemokine levels

Cord blood was collected by needle puncture from the umbilical cord vein at birth and separated serum was frozen at -80 degree until use. Chemokine measurement was performed simultaneously for the Bio-Plex Human chemokine, 4-plex assay kit (Bio-Rad Laboratories, Hercules, CA), namely IP-10/CXCL10, I-TAC/CXCL11, MDC/CCL22 and TRAC/CCL17, according to the manufacturer's instructions. Briefly, 50 ul of sample was incubated with antibody-coupled beads and biotinylated detection antibodies at room temperature. The beads were eventually re-suspended in 125 ul assay buffer and read on the Bio-Plex suspension array system. Data were analyzed using Bio-Plex Manager software version 6.0. The limit of detection was 1.6 pg/mL for CXCL10, 0.1 pg/mL for CXCL11, 0.9 pg/mL for CCL22 and 1.7 pg/mL for CCL17. All samples were analyzed in duplicates with the coefficient of variation (CV) < 10%.

### Covariates

Confounding variables associated with the development of atopic diseases were included and adjusted in the multiple logistic regression analysis. Confounders including child's sex, gestational age, and maternal age at delivery, maternal history of atopy, any passive smoke exposure during pregnancy, any older siblings at birth and household income were collected and analyzed.

### Statistical analysis

Comparisons of baseline characteristics of children between groups were made using univariate parametric and non-parametric tests such as Student t test, c2, and Fisher's exact test. The differences between continuous variables with non-normal distribution were estimated with the Mann-Whitney test. Standard binary logistic regression analysis methods were used to analyze the association between cord blood chemokines (CCL22, CCL17, CXCL10 and CXCL11) and IgE sensitization or allergen sensitization. Receiver operating characteristic (ROC) analysis was employed for the significant continuous variables and the area under the ROC significantly different from 0.5 was categorized with the cutoff values. Multiple logistic regression analysis was used to determine the associations of cord blood Th1/Th2 chemokines and allergic sensitization and atopic diseases by adjusting the confounders related to atopic diseases. Statistical analysis was performed using the Statistical Package for the Social Sciences (SPSS Statistics for Windows Version 20.0; Armonk, NY, USA). All statistical hypothesis tests were two-tailed and *P*-values < 0.05 were considered significant.

## References

[1] Zhang Y, Gu W, He L, Sun B (2014). Th1/Th2 cell's function in immune system. Adv Exp Med Biol.

[2] Joos L, IE Carlen Brutsche, Laule-Kilian K, Crawen M, Tamm M, Brutsche MH (2004). Systemic Th1- and Th2-gene signals in atopy and asthma. Swiss Med Wkly.

[3] Cook-Mills JM (2015). Maternal influences over offspring allergic responses. Curr Allergy Asthma Rep.

[4] Abelius MS, Lempinen E, Lindblad K, Ernerudh J, Berg G, Matthiesen L, Nilsson LJ, Jenmalm MC (2014). Th2-like chemokine levels are increased in allergic children and influenced by maternal immunity during pregnancy. Pediatr Allergy Immunol.

[5] Nakazato J, Kishida M, Kuroiwa R, Fujiwara J, Shimoda M, Shinomiya N (2008). Serum levels of Th2 chemokines, CCL17, CCL22, and CCL27, were the important markers of severity in infantile atopic dermatitis. Pediatr Allergy Immunol.

[6] Chiu CY, Tsai MH, Yao TC, Tu YL, Hua MC, Yeh KW, Huang JL (2014). Urinary LTE4 levels as a diagnostic marker for IgE-mediated asthma in preschool children: a birth cohort study. PLoS One.

[7] Chiu CY, Huang YL, Tsai MH, Tu YL, Hua MC, Yao TC, Yeh KW, Huang JL (2014). Sensitization to food and inhalant allergens in relation to atopic diseases in early childhood: a birth cohort study. PLoS One.

[8] Kool M, Hammad H, Lambrecht BN (2012). Cellular networks controlling Th2 polarization in allergy and immunity. F1000. Biol Rep.

[9] Nomura T, Terada N, Kim WJ, Nakano K, Fukuda Y, Wakita A, Numata T, Konno A (2002). Interleukin-13 induces thymus and activation-regulated chemokine (CCL17) in human peripheral blood mononuclear cells. Cytokine.

[10] Cole KE, Strick CA, Paradis TJ, Ogborne KT, Loetscher M, Gladue RP, Lin W, Boyd JG, Moser B, Wood DE, Sahagan BG, Neote K (1998). Interferon-inducible T cell alpha chemoattractant (I-TAC): a novel non-ELR CXC chemokine with potent activity on activated T cells through selective high affinity binding to CXCR3. J Exp Med.

[11] Folsgaard NV, Chawes BL, Bonnelykke K, Jenmalm MC, Bisgaard H (2012). Cord blood Th2-related chemokine CCL22 levels associate with elevated total-IgE during preschool age. Clin Exp Allergy.

[12] Manry J, Laval G, Patin E, Fornarino S, Itan Y, Fumagalli M, Sironi M, Tichit M, Bouchier C, Casanova JL, Barreiro LB, Quintana-Murci L (2011). Evolutionary genetic dissection of human interferons. J Exp Med.

[13] Leung TF, Wong GW, Ko FW, Lam CW, Fok TF (2004). Increased macrophage-derived chemokine in exhaled breath condensate and plasma from children with asthma. Clin Exp Allergy.

[14] Abelius MS, Ernerudh J, Berg G, Matthiesen L, Nilsson LJ, Jenmalm MC (2011). High cord blood levels of the T-helper 2-associated chemokines CCL17 and CCL22 precede allergy development during the first 6 years of life. Pediatr Res.

[15] Sun BQ, Zheng PY, Zhang XW, Huang HM, Chen DH, Zeng GQ (2014). Prevalence of allergen sensitization among patients with allergic diseases in Guangzhou, Southern China: a four-year observational study. Multidiscip Respir Med.

[16] Abrahamsson TR, M Sandberg Abelius, Forsberg A, Bjorksten B, Jenmalm MC (2011). A Th1/Th2-associated chemokine imbalance during infancy in children developing eczema, wheeze and sensitization. Clin Exp Allergy.

[17] Yao TC, Ou LS, Yeh KW, Lee WI, Chen LC, Huang JL (2011). Associations of age, gender, and BMI with prevalence of allergic diseases in children: PATCH study. J Asthma.

[18] Chiu CY, Yao TC, Chen SH, Tsai MH, Tu YL, Hua MC, Yeh KW, Huang JL (2014). Low cord blood vitamin D levels are associated with increased milk sensitization in early childhood. Pediatr Allergy Immunol.

[19] Seymour JL, Keswick BH, Hanifin JM, Jordan WP, Milligan MC (1987). Clinical effects of diaper types on the skin of normal infants and infants with atopic dermatitis. J Am Acad Dermatol.

[20] Togias AG (2000). Systemic immunologic and inflammatory aspects of allergic rhinitis. J Allergy Clin Immunol.

[21] Yao TC, Tu YL, Chang SW, Tsai HJ, Gu PW, Ning HC, Hua MC, Liao SL, Tsai MH, Chiu CY, Lai SH, Yeh KW, Huang JL (2014). Suboptimal vitamin D status in a population-based study of Asian children: prevalence and relation to allergic diseases and atopy. PLoS One.

[22] Gergen PJ, Turkeltaub PC, Kramer RA (1992). Age of onset in childhood asthma: data from a national cohort. Ann Allergy.

[23] Sunyer J, Anto JM, Castellsague J, Soriano JB, Roca J (1996). Total serum IgE is associated with asthma independently of specific IgE levels. The Spanish Group of the European Study of Asthma. Eur Respir J.

[24] Lee AJ, Thalayasingam M, Lee BW (2013). Food allergy in Asia: how does it compare?. Asia Pac Allergy.

[25] Ballardini N, Nilsson C, Nilsson M, Lilja G (2006). ImmunoCAP Phadiatop Infant—a new blood test for detecting IgE sensitisation in children at 2 years of age. Allergy.

